# Emergency Department Pre-Viability Delivery of a Fetus En Caul

**DOI:** 10.7759/cureus.22338

**Published:** 2022-02-17

**Authors:** Margaret Moran

**Affiliations:** 1 Emergency Medicine, Brooke Army Medical Center, Fort Sam Houston, USA

**Keywords:** end of life care, emergency department, obstetrics, en caul delivery, precipitous delivery

## Abstract

En caul is a term used to describe the birth of a fetus and placenta entirely encased in an un-ruptured amniotic sac. Also colloquially referred to as a “mermaid” or “veiled” delivery, en caul births are uncommon in obstetrics literature and, therefore, exceedingly rare in the emergency department. Here, the author presents a case of a 34-year-old female with pre-viability delivery of a fetus en caul in the emergency department. Pre-viability delivery, regardless of membrane intactness, is associated with inevitable fetal loss. While emergency department physicians look for actionable practice guidelines, obstetric literature espouses expectant management, which consists of patient education and shared decision-making regarding patient comfort and goals of care. The author will discuss key diagnostic modalities and management steps for emergency department delivery of extremely premature infants.

## Introduction

En caul delivery, commonly referred to as “mermaid birth” or “veiled birth”, describes the delivery of an intact amnion. While en caul delivery is rare in obstetrics literature, occurring in less than 1 in 80,000 vaginal deliveries, it can be inferred that emergency department delivery of an en caul fetus is exceedingly rare [1]. Pre-viability delivery, regardless of membrane intactness, is associated with inevitable fetal loss [2]. It can be difficult for emergency medicine physicians, who specialize in decision making and acting on decisions to prevent further morbidity and mortality [3], to decide on a course of inaction, namely expectant management. On literature review, en caul delivery is most commonly associated with cesarean delivery and extreme prematurity [4]. The gestational age of viability has progressed to nearly 22 weeks with advances in perinatology and neonatology, however, delivery below 22 weeks results in inevitable mortality [5]. Pregnancy loss and fetal demise are emotionally charged diagnoses for patients and physicians alike. A familiarity with diagnosis, management, and expected clinical course can ease discomfort for providers and allow for clearer communication of expectations for patients.

## Case presentation

A 34-year-old female gravida 3 para 2 at approximately 19 weeks and six days by dates presented to the emergency department, brought in by emergency medical services (EMS), for evaluation of persistent pelvic pressure since urinating earlier this morning. The patient reported intermittent, cramping, pelvic pain which did not improve with recumbency or oral hydration. EMS report included the phrase “amniotic membranes intact”.

**Figure 1:**
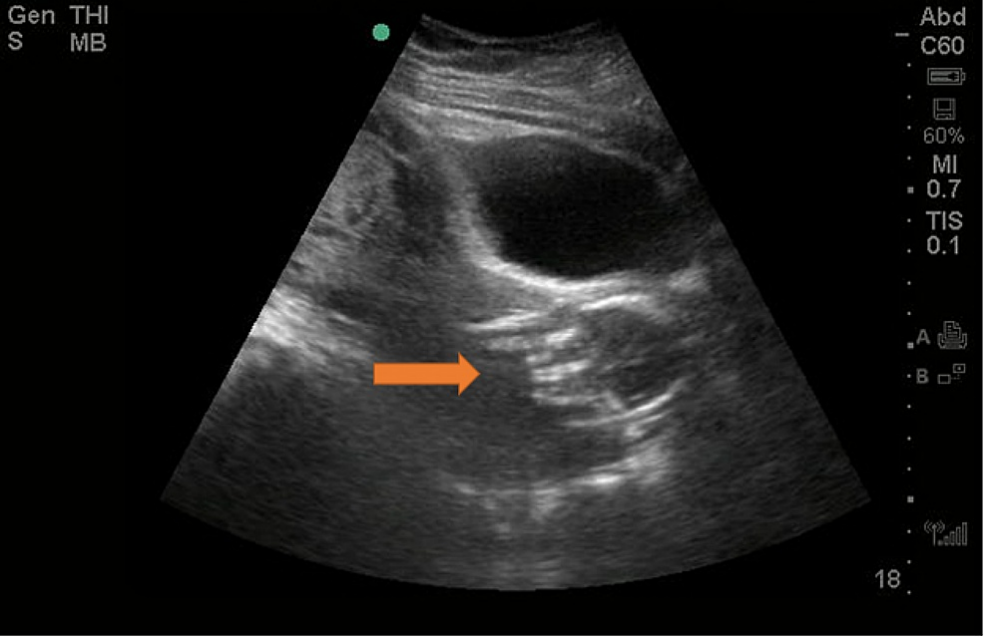
Intrauterine fetus without surrounding amniotic fluid (red arrow).

Initial vital signs included a blood pressure of 117/59 mmHg, heart rate of 92 beats per minute, respiratory rate of 20 breaths per minute, oxygen saturation of 96% on room air, and a temperature of 98.2°F. Physical exam was notable for a soft, non-tender, non-distended abdomen without rebound or guarding. External genital exam demonstrated bulging membranes from the vaginal introitus. The patient’s amniotic sac was full of clouded, dark fluid with grossly visible swirling sediment. Bedside ultrasonography (Figure 1) revealed a single, living intrauterine fetus with a fetal heart rate of 116 beats per minute without surrounding amniotic fluid with detectable fetal movement. Again, at the vaginal introitus, a tense amniotic sac is noted.

Obstetrics service was consulted and with the reported history of cloudy, dark amniotic fluid, leading differential diagnosis was chorioamnionitis. Antibiotics were administered. Given partial expulsion of the amniotic sac in conjunction with gestational age, obstetrics recommended expectant management.

While in the emergency department, prior to obstetrics arrival, the patient reported an urge to “push”. Repeat ultrasonography overlying the suprapubic region revealed a fetus without cardiac activity. After several minutes, the patient expelled a mass of tissue from her vaginal introitus.

## Discussion

First trimester vaginal bleeding or discomfort is an extremely common chief complaint in the emergency department. Twenty to 40% of pregnant persons will experience vaginal bleeding in the first trimester and many of these persons will choose to be evaluated in the emergency department for their concerns [6]. A survey including data from 1993 to 2003 demonstrated that vaginal bleeding in early pregnancy accounted for 1.6% of all emergency department visits during this time period, or nearly 500,000 visits. The authors of this survey also concluded that emergency department visits for vaginal bleeding in early pregnancy are on the rise [7]. The evaluation of second trimester complaints in the emergency department is comparatively rare. A majority of pregnant patients presenting to the emergency department for evaluation of vaginal bleeding in early pregnancy are seeking evaluation of the well-being of their fetus [8]. Fetal heart rate over 110 beats per minute in conjunction with visible fetal movement and surrounding amniotic fluid on ultrasonography is reassuring [9]. As in the presented case, clinical context is important in determining fetal well-being. It can be emotionally difficult for both providers and patients when the well-being of the fetus is in jeopardy. When intact amniotic membranes are observed in the vaginal introitus, initial instinct may be to replace the intact amnion inside the uterus, however, replacement is never a viable pathway as infection risk to the pregnant patient is great. It is within the purview of emergency medicine providers to manage extreme preterm precipitous delivery in the emergency department [10]. En caul presentation adds an extra layer of complexity, however, management of extreme preterm precipitous delivery is the same, regardless of membrane rupture. Once it has been determined that a pregnancy is no longer viable, there are several management strategies including medical, surgical, and expectant management.

Expectant management involves nonintervention, allowing a pregnancy to progress as expected, including spontaneous labor [10]. Expectant management of a premature fetus can be difficult for the emergency medicine physician, in particular, when it occurs at an age of peri-viability. In extreme pre-term labor, defined as less than 26 weeks gestation, expectant management results in vaginal delivery, on average, four days after admission, which is a significantly longer timeline than the average emergency medicine physician practices within [11]. Expectant management can be a difficult pathway for emergency medicine providers, who specialize in decision making and action taking in critical patient encounters. Once a physician has determined that expectant management will result in imminent delivery, care should be taken to discuss expected clinical course with your patient and any support persons. Although rare, it is important for emergency physicians to remember that fetal demise in the second trimester may present to the emergency department as extreme pre-term labor and delivery.

## Conclusions

Imminent extreme premature delivery can be an emotionally fraught patient encounter for emergency providers, made even more complex in rare presentations, such as en caul. Familiarity with this rare presentation can help direct expectations and resources to facilitate the best possible outcome, which may not be maintaining viability. This includes awareness of diagnostic modalities and management. En caul delivery in isolation does not portend increased morbidity or mortality, however, en caul delivery is most commonly observed in extreme prematurity. Management of extreme premature delivery in the emergency department is unchanged regardless of amniotic membrane rupture.
